# Nitric oxide signalling in roots is required for MYB72-dependent systemic resistance induced by *Trichoderma* volatile compounds in Arabidopsis

**DOI:** 10.1093/jxb/erab294

**Published:** 2021-06-15

**Authors:** Leyre Pescador, Iván Fernandez, María J Pozo, María C Romero-Puertas, Corné M J Pieterse, Ainhoa Martínez-Medina

**Affiliations:** Department of Soil Microbiology and Symbiotic Systems, Estación Experimental del Zaidín (CSIC), Profesor Albareda 1, 18008 Granada, Spain; Department of Biochemistry, Cell and Molecular Plant Biology, Estación Experimental del Zaidín (CSIC), Profesor Albareda 1, 18008 Granada, Spain; Molecular Interaction Ecology, German Centre for Integrative Biodiversity Research (iDiv) Halle-Jena-Leipzig/Institute of Biodiversity, Friedrich Schiller University Jena, Puschstraße 4, 04103 Leipzig, Germany; Plant–Microorganism Interaction Research Group, Institute of Natural Resources and Agrobiology of Salamanca (IRNASA-CSIC), Cordel de Merinas 40, 37008 Salamanca, Spain; Department of Soil Microbiology and Symbiotic Systems, Estación Experimental del Zaidín (CSIC), Profesor Albareda 1, 18008 Granada, Spain; Department of Biochemistry, Cell and Molecular Plant Biology, Estación Experimental del Zaidín (CSIC), Profesor Albareda 1, 18008 Granada, Spain; Plant-Microbe Interactions, Department of Biology, Utrecht University, Padualaan 8, 3584 CH Utrecht, The Netherlands; Plant–Microorganism Interaction Research Group, Institute of Natural Resources and Agrobiology of Salamanca (IRNASA-CSIC), Cordel de Merinas 40, 37008 Salamanca, Spain; University of Edinburgh, UK

**Keywords:** Arabidopsis, defence priming, induced systemic resistance, microbial volatile compounds, MYB72, nitric oxide, *Trichoderma*

## Abstract

Volatile compounds (VCs) of *Trichoderma* fungi trigger induced systemic resistance (ISR) in Arabidopsis that is effective against a broad spectrum of pathogens. The root-specific transcription factor MYB72 is an early regulator of ISR and also controls the activation of iron-deficiency responses. Nitric oxide (NO) is involved in the regulation of MYB72-dependent iron-deficiency responses in Arabidopsis roots, but the role of NO in the regulation of MYB72 and ISR by *Trichoderma* VCs remains unexplored. Using *in vitro* bioassays, we applied *Trichoderma* VCs to Arabidopsis seedlings. Plant perception of *Trichoderma* VCs triggered a burst of NO in Arabidopsis roots. By suppressing this burst using an NO scavenger, we show the involvement of NO in *Trichoderma* VCs-mediated regulation of *MYB72* expression. Using an NO scavenger and the Arabidopsis lines *myb72* and *nia1nia2* in *in planta* bioassays, we demonstrate that NO signalling is required in the roots for activation of *Trichoderma* VCs-mediated ISR against the leaf pathogen *Botrytis cinerea*. Analysis of the defence-related genes *PR1* and *PDF1.2* points to the involvement of root NO in priming leaves for enhanced defence. Our results support a key role of root NO signalling in the regulation of *MYB72* expression during the activation of ISR by *Trichoderma* VCs.

## Teaser

Nitric oxide signalling in roots is required for induced systemic resistance by *Trichoderma* volatile compounds in leaves by regulating the root-specific transcription factor MYB72 in Arabidopsis.

## Introduction

Plant roots host a plethora of soil microbes that can establish beneficial interactions (Berendsen *et al.*, 2012). Among them, plant interaction with fungi from the genus *Trichoderma* (hereafter Trichoderma) provides essential services to the plant, improving plant nutrition and protection against soil-borne pathogens (Harman *et al.*, 2004; Viterbo and Horwitz, 2010; Harman, 2011; Hermosa *et al.*, 2012). Moreover, selected Trichoderma isolates can confer a form of systemic immunity in their host that is effective against a broad spectrum of shoot and root pathogens, a phenomenon known as induced systemic resistance (ISR) (Martínez-Medina *et al*., 2013, 2017*a*; Pieterse *et al.*, 2014). ISR can also be conferred by other beneficial microbes, such as plant growth-promoting rhizobacteria and mycorrhizal fungi (Van Wees *et al.*, 2008; Shoresh *et al.*, 2010; Jung *et al.*, 2012; Pieterse *et al.*, 2014). Typically, ISR triggered by beneficial microbes, including Trichoderma fungi, is associated with priming of the plant immune system, resulting in an enhanced and/or faster activation of plant defences upon pathogen attack (Van Wees *et al.*, 2008; Martínez-Medina *et al*., 2013, 2017*a*; Mauch-Mani *et al.*, 2017). Defence priming by beneficial microbes provides the plant with a cost-effective mechanism of protection against shoot and root attackers (Martínez-Medina *et al*., 2016; Mauch-Mani *et al.*, 2017).

The *Arabidopsis thaliana* root R2R3-type MYB transcription factor MYB72 is an essential regulator of the initiation of ISR mediated by beneficial microbes, including Trichoderma fungi. Arabidopsis *myb72* mutant plants are impaired in their ability to express ISR triggered by *Trichoderma asperellum* root colonization (Segarra *et al.*, 2009). Interestingly, ISR mediated by *Pseudomonas simiae* WCS417 (formerly known as *Pseudomonas fluorescens*; Berendsen *et al.*, 2015; Pieterse *et al.*, 2021) is also dependent on MYB72 (Van der Ent *et al.*, 2008), indicating that this transcription factor is a node of convergence in the ISR signalling pathways triggered by different beneficial microbes. Besides regulating the onset of ISR in roots, MYB72 has been shown to control the biosynthesis and excretion of iron-mobilizing coumarins in the root environment (Zamioudis *et al.*, 2014; Stringlis *et al.*, 2018). Specific MYB72-dependent coumarins have selective antimicrobial activity and play a role in shaping root microbiome assembly to promote plant growth and health (Stringlis *et al.* 2018). In addition to MYB72, signalling molecules, such as the hormones jasmonic acid (JA), salicylic acid (SA), ethylene, and abscisic acid, have been implicated in Trichoderma-mediated ISR (Martínez-Medina *et al.*, 2013; Saravanakumar *et al.*, 2016; Alkooranee *et al.*, 2017; Agostini *et al.*, 2019). More recently, the signalling molecule nitric oxide (NO) has been suggested to be further involved in ISR mediated by Trichoderma in cucumber plants (Nawrocka *et al.*, 2019), although its specific role in Trichoderma-mediated ISR remains obscure.

NO is a highly reactive free radical that can diffuse across biological membranes due to its gaseous and lipophilic nature and can be a counterpart of cell-to-cell signalling over short periods of time (Beligni and Lamattina, 2001; Brouquisse, 2019; León and Costa-Broseta, 2019). NO is involved in a wide range of plant processes, such as seed germination (Albertos *et al.*, 2015), root development (Sanz *et al.*, 2015), and plant reproduction (Du *et al.*, 2014). NO is also implicated in plant responses to several abiotic and biotic stresses, including adaptation to low iron availability (Graziano and Lamattina, 2007; Chen *et al.*, 2010; García *et al.*, 2010; Meiser *et al.*, 2011) and defence responses against pathogen attack (Martínez-Medina *et al.*, 2019*c*; Molina-Moya *et al.*, 2019). NO has been shown to be further involved in plant interaction with beneficial microbes (Meilhoc *et al.*, 2013; Berger *et al*., 2019, 2020; Martínez-Medina *et al*., 2019*a*, *b*). NO accumulates rapidly in the roots of Arabidopsis and tomato plants during interactions with Trichoderma fungi, suggesting a role for NO in the establishment of the plant–Trichoderma symbiosis (Gupta *et al.*, 2014; Martínez-Medina *et al.*, 2019*a*).

We recently found that volatile compounds (VCs) from the ISR-inducing Trichoderma fungi *Trichoderma harzianum* T-78 and *Trichoderma asperellum* T-34 act as determinants of the elicitation of ISR against the necrotrophic fungus *Botrytis cinerea* (Martínez-Medina *et al.*, 2017*b*). Moreover, we demonstrated that root perception of Trichoderma VCs triggered the expression of *MYB72* as part of the activation of the strategy I response to iron deficiency in Arabidopsis roots. Similarly, VCs released by the ISR-inducing rhizobacteria *P. simiae* WCS417 triggered the expression of *MYB72* in Arabidopsis roots (Zamioudis *et al.*, 2015), indicating that elicitation of *MYB72* expression and activation of the strategy I iron uptake response is a feature conserved among different root-associated mutualists. Interestingly, elicitation of *MYB72* expression by rhizobacterial VCs in Arabidopsis roots was found to be associated with NO signalling (Zamioudis *et al.* 2015), indicating that NO might act upstream of MYB72 in the activation of ISR and the strategy I iron uptake response mediated by microbial VCs.

Here we hypothesize that NO signalling in the roots is an early key component in the Trichoderma-mediated activation of *MYB72* expression and ISR in Arabidopsis. To test this hypothesis, we first monitored NO accumulation in Arabidopsis roots in response to exposure to Trichoderma VCs. We found that plant perception of Trichoderma VCs triggered an early accumulation of NO in Arabidopsis roots. By using the NO scavenger 2-(4 carboxyphenyl)-4,4,5,5-tetramethylimidazoline-1-oxyl-3-oxide (cPTIO), we showed that NO-dependent signalling is involved in Trichoderma VCs-mediated induction of *MYB72* in Arabidopsis roots. Moreover, by performing different bioassays, including the use of the NO scavenger in combination with the Arabidopsis *myb72* mutant and the *nia1nia2* mutant, which is impaired in nitrate reductase-dependent NO production, we demonstrated the requirement for NO signalling in roots during the activation of MYB72-dependent ISR against the leaf pathogen *B. cinerea* triggered by Trichoderma VCs. Collectively, our results reveal an important role of NO signalling in roots in the regulation of *MYB72* expression during the activation of ISR triggered by Trichoderma VCs.

## Materials and methods

### Plant and fungal material

We used *A. thaliana* wild-type (WT) accession Col-0, the Arabidopsis *myb72-2* mutant line (Van der Ent *et al.*, 2008), the Arabidopsis reporter line *pMYB72:GFP-GUS* (Zamioudis *et al.*, 2015) and the Arabidopsis *nia1nia2* double mutant, which is impaired in nitrate reductase-dependent NO production (Wilkinson and Crawford, 1993). Arabidopsis seeds were surface disinfected and stratified for 2 days at 4 °C. *Trichoderma harzianum* T-78 (T-78; Martínez-Medina *et al.*, 2013) and *T. asperellum* T-34 (T-34; Segarra *et al.*, 2009) were cultured on potato dextrose agar plates for 5 days at 28 °C. *Botrytis cinerea* strain B05.10 (Van Kan *et al.*, 1997) was cultivated on half-strength potato dextrose agar plates for 10 days at 22 °C.

### Bioassays in plates

Surface-sterilized Arabidopsis seeds were sown on Murashige and Skoog (MS) agar-solidified medium supplemented with vitamins and 0.5% sucrose, pH 6, in one of the compartments of two-compartment circular plates (120 mm diameter), according to Zamioudis *et al.* (2015) and Martínez-Medina *et al.* (2017b). The plates were positioned vertically and placed in a growth chamber (22 °C, 10 h light/14 h dark, light intensity 100 μmol m^–2^ s^–1^). After 12 days, a 7 mm diameter plug of each Trichoderma strain from the actively growing margins of cultures was transferred into the plant-free compartment containing MS agar-solidified medium. The plates were sealed with one layer of gas-permeable Parafilm (Sigma) and placed in a vertical position in the growth chamber for 1, 2, or 3 days. In the two-compartment plates, the seedlings and microbes were physically separated, but gas exchange was allowed between the compartments.

### Bioassays in pots

Individual seedlings that had been growing in the plates for 15 days were transferred to 50 ml pots containing sterile sand:soil mixture (5:12, v:v). Plants were then randomly distributed and cultivated in a growth chamber with an 8 h light (24 °C, light intensity 100 μmol m^–2^ s^–1^) and 16 h dark (20 °C) cycle at 70% relative humidity. Plants were watered every other day and received half-strength Hoagland solution (Hoagland and Arnon, 1938) containing 10 μM Sequestrene (CIBA-Geigy) once a week.

### *Botrytis cinerea* bioassays

Five-week-old Arabidopsis plants were inoculated with *B. cinerea* strain B05.10 (Van Kan *et al.*, 1997) according to Van Wees *et al.* (2013). A 5 μl droplet of a suspension of 5×10^5^ spores ml^–1^ was applied to six leaves of each plant. Thereafter, plants were placed under a lid to increase the relative humidity to 100% to promote the infection. Disease symptoms were scored 3 days after *B. cinerea* inoculation by visual inspection. Disease ratings were assigned to each leaf according to Van der Ent *et al.* (2008), and the percentage of leaves in each disease severity class was calculated for each plant. Shoot samples for quantification of *B. cinerea TUBULIN* mRNA levels were harvested 1 day after *B. cinerea* inoculation.

### Fluorescence microscopy

The presence of NO in Arabidopsis roots was analysed using the cell-permeable NO-specific probe 4,5-diaminofluorescein diacetate (DAF-2DA), which is converted to its fluorescent triazole derivative DAF-2T upon reaction with NO (Nakatsubo *et al.*, 1998). Segments of plant primary roots from the apex were incubated for 1 h in darkness with 10 µM DAF-2DA (Merck Biosciences), prepared in 10 mM Tris–HCl (pH 7.4) as described by Sandalio *et al.* (2008). As a negative control, root segments were similarly incubated with the NO scavenger cPTIO (Sigma) at a final concentration of 500 µM. Subsequently, the segments were washed three times for 15 min each in 10 mM Tris–HCl (pH 7.4). The fluorescence emitted by DAF-2T was detected by excitation at 495 nm and emission at 515 nm (Sandalio *et al.*, 2008). Fluorescence intensity was quantified by using ZEN Lite software (Zeiss). As counterstain, roots were stained with 10 μg ml^–1^ propidium iodide solution for 2 min. Green fluorescent protein (GFP) fluorescence in *pMYB72:GFP-GUS* was examined on a Leica MZ16FA fluorescence stereomicroscope equipped with a GFP3 filter.

### Chemical treatment

To study whether NO is required for the regulation of *MYB72* expression by Trichoderma VCs, inhibition of NO signalling was achieved by transferring seedlings to plates containing MS agar-solidified medium supplemented with the NO scavenger cPTIO at a final concentration of 500 µM (Terrón-Camero *et al.*, 2020). To study whether NO is required for Trichoderma VCs-mediated ISR against *B. cinerea*, roots of Arabidopsis plants growing in split plates were treated with 500 µl of 500 µM cPTIO. The cPTIO was applied every 8 h for the duration of the split-plate bioassay. For control plates, the same procedure was done with sterile water.

### Real-time quantitative RT–PCR

Total RNA from Arabidopsis leaves was extracted using Tri-sure (Bioline) and subsequently purified using the RNA Clean and Concentrator-5 kit (Zymo Research). RNA samples were treated with NZY DNase I (NZYTech). First-strand cDNA was synthesized from 1 μg of purified total RNA by using the PrimeScript RT Master Mix (Takara). Real-time quantitative PCR (RT–qPCR) reactions were performed using SYBR®Premix Ex Taq^TM^ (Takara) and an iCycler 5 (Bio-Rad). All kits were used according to the manufacturers’ instructions. Relative quantification of specific mRNA levels was performed using the comparative 2^–ΔΔCt^ method (Livak and Schmittgen, 2001) by using the gene-specific primers described in Supplementary Table S1. Expression values were normalized using the Arabidopsis housekeeping genes *TUBULIN-4* (At5g44340) or *ACTIN7* (At5g09810). Fungal infection was measured by analysing the *B. cinerea β-TUBULIN* gene (XM_001560987.1) relative to the Arabidopsis *TUBULIN-4* gene.

## Results

### MYB72 is required for Trichoderma VCs-mediated ISR

**Fig. 1.**
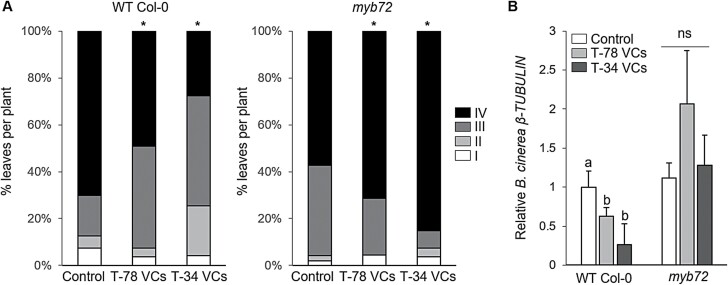
MYB72 is required for Trichoderma VCs-triggered systemic immunity. (A) Quantification of *B. cinerea* disease symptoms and (B) relative amount of *B. cinerea* in leaves of Arabidopsi*s* WT Col-0 and *myb72* mutant lines after inoculation with *B. cinerea*. Seedlings were untreated (control) or treated with VCs from *T. harzianum* T-78 (T-78 VCs) or *T. asperellum* T-34 (T-34 VCs) for 3 days in split-plate assays before being transplanted into pots. Three weeks after transplanting, the seedlings were challenged with *B. cinerea*. In (A), disease severity was scored 3 days after inoculation by using four disease severity classes: I, no visible disease symptoms; II, non-spreading lesion; III, spreading lesion without tissue maceration; IV, spreading lesion with tissue maceration and sporulation of the pathogen. The percentage of leaves in each class was calculated per plant. Asterisks indicate statistically significant differences compared with untreated control plants (χ ^2^ test; α=0.05; *n*=50 plants). In (B), the relative amount of *B. cinerea* was determined 1 day after inoculation by qRT–PCR analysis of the *B. cinerea β-TUBULIN* gene relative to the Arabidopsis *TUBULIN-4* gene. The expression levels are reported as the fold change relative to that found in control Col-0 plants. Values are the means ±SE of four biological replicates. For each Arabidopsis line, different letters indicate statistically significant differences between treatments (Tukey’s test; *P*<0.05). ns, not significant.

**Fig. 2.**
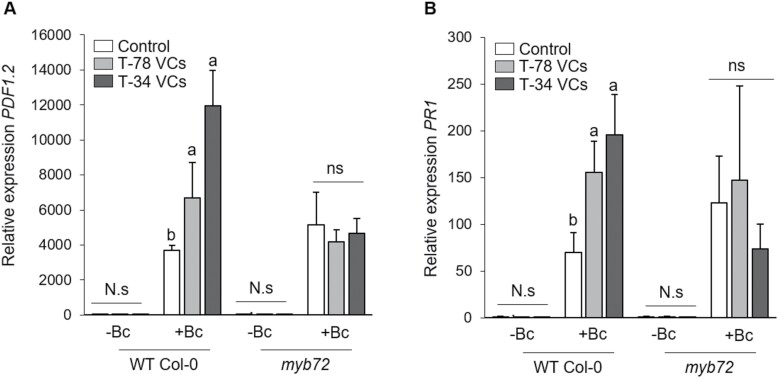
Role of MYB72 in Trichoderma VCs-mediated priming for enhanced defence-related gene expression in response to *B. cinerea* infection. Relative expression of the defence-related marker genes *PDF1.2* (A) and *PR1* (B) in leaves of Arabidopsi*s* WT Col-0 and *myb72* mutant lines, 1 day after inoculation with *B. cinerea*. Seedlings were untreated (control) or treated with VCs from *T. harzianum* T-78 (T-78 VCs) or *T. asperellum* T-34 (T-34 VCs) for 3 days in split-plate assays before transplanting them into pots. Three weeks after transplanting, seedlings were inoculated with *B. cinerea* (+Bc) or remained uninoculated (-Bc). Expression levels were normalized to that of the Arabidopsis *TUBULIN-4* gene. For each gene, the expression levels are reported as the fold increase relative to that found in control Col-0 plants not inoculated with *B. cinerea*. Values are the means ±SE of four biological replicates. For each Arabidopsis line, different letters in uninoculated (-Bc) and inoculated (+Bc) plants indicate statistically significant differences between treatments (Tukey’s test; *P*<0.05). ns, not significant.

We previously found that VCs released by T-78 and T-34 trigger ISR against the shoot pathogen *B. cinerea* (Martínez-Medina *et al.*, 2017*b*). The transcription factor MYB72 is essential for the onset of the ISR mediated by root colonization of beneficial rhizobacteria and rhizofungi (Van der Ent *et al.*, 2008; Segarra *et al.*, 2009). Here, we first aimed to investigate whether MYB72 is also required for Trichoderma VCs-mediated ISR. To this end, Arabidopsis Col-0 and *myb72* seedlings were treated with VCs from T-78 or T-34, or used as untreated controls, for 3 days in split-plate assays. Treatment with Trichoderma VCs enhanced the expression of the Arabidopsis *FRO2* (*FERRIC REDUCTION OXIDASE2*) and *IRT1* (*FE-REGULATED TRANSPORTER1*) genes in the roots of WT Col-0 and *myb72* seedlings (Supplementary Fig. S1), confirming previous findings (Martínez-Medina *et al.*, 2017*b*). After treatment with VCs, Col-0 and *myb72* seedlings were transplanted into pots, and 3 weeks later the plants were challenged with the necrotrophic pathogen *B. cinerea*. We observed that Col-0 plants that had previously been exposed to T-78 or T-34 VCs developed significantly less severe disease symptoms than untreated control plants (Fig. 1A) and contained less pathogen biomass, as determined by qRT–PCR quantification of the constitutively expressed *B. cinerea* gene *β-TUBULIN* (Fig. 1B). In addition, upon pathogen infection we found stronger expression of the JA-responsive gene *PDF1.2* (*PLANT DEFENSIN1.2*; Penninckx *et al.*,1998) (Fig. 2A) and the SA-responsive marker gene *PR1* (*PATHOGENESIS-RELATED PROTEIN 1*; Uknes *et al.*, 1992) (Fig. 2B) in Col-0 plants previously treated with fungal VCs compared with untreated (control) plants. Remarkably, in the absence of *B. cinerea* infection, treatments with T-78 or T-34 VCs were not associated with transcriptional activation of *PDF1.2* and *PR1* (Fig. 2A, B), showing that T-78 and T-34 VCs prime Arabidopsis seedlings for enhanced JA- and SA-responsive gene expression in the shoots. Interestingly, *myb72* plants failed to mount a VCs-mediated ISR, as the *myb72* plants treated with fungal VCs developed more severe symptoms than untreated *myb72* plants (Fig. 1A). Moreover, treatment of *myb72* plants with fungal VCs did not reduce *B. cinerea* biomass (Fig. 1B) and did not boost *PDF1.2* and *PR1* expression upon pathogen infection (Fig. 2A, B). These results show that, in analogy to the ISR triggered by Trichoderma root inoculation (Supplementary Fig. S2), the defence priming and ISR response mediated by Trichoderma VCs requires MYB72.

### Trichoderma VCs trigger the accumulation of NO in Arabidopsis roots

**Fig. 3.**
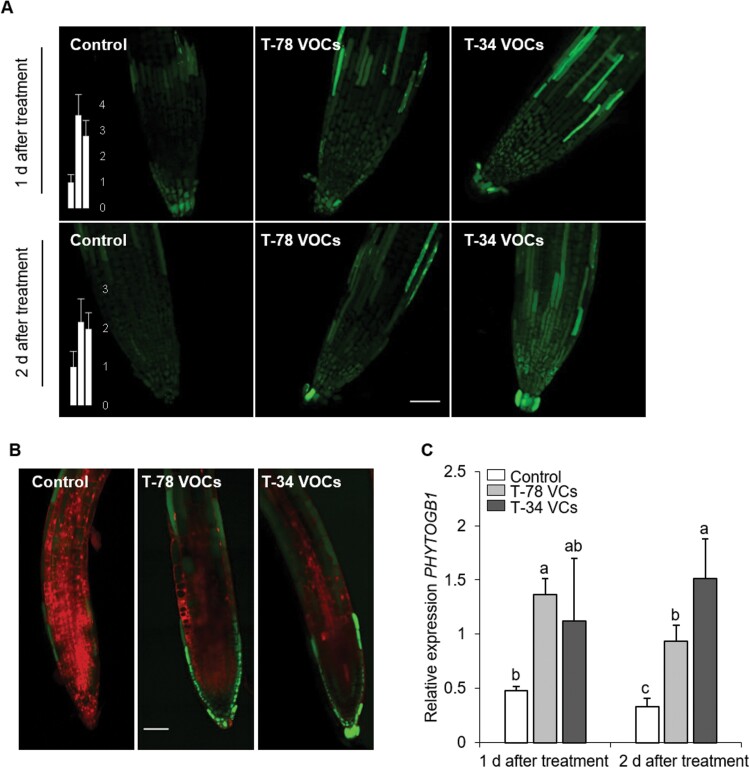
Trichoderma VCs trigger NO accumulation in Arabidopsis roots. (A) Imaging of NO production in roots of Arabidopsis Col-0 seedlings by confocal laser scanning fluorescence microscopy. Images are projections of several optical sections collected by confocal microscopy showing NO-dependent DAF-2DA fluorescence (green; excitation at 495 nm, emission at 515 nm) from untreated (control) seedlings, or seedlings treated with VCs from *T. harzianum* T-78 (T-78 VCs) or *T. asperellum* T-34 (T-34 VCs) for 1 or 2 days using split-plate assays. Scale bar=50 μm. Bars show the relative fluorescence intensities corresponding to NO, quantified in arbitrary units in untreated controls (left bar), seedlings treated with T-78 VCs (middle bar) or T-34 VCs (right bar). (B) Representative confocal laser scanning fluorescence microscopy images of NO accumulation in roots of Arabidopsis seedlings untreated (control) or treated with VCs from T-78 or T-34 for 1 day. The images show DAF-2DA fluorescence as in (A). Cell walls were counterstained with propidium iodide (red signal). Scale bar=50 μm. These results are representative of two independent experiments. (C) Expression of the NO-responsive gene *PHYTOGB1* in Arabidopsis roots. Seedlings were either untreated (control) or treated with VCs from T-78 or T-34 for 1 or 2 days. Results were normalized to the *ACTIN7* gene expression in the same samples. Values are the means ±SE of five biological replicates; each biological replicate consisted of pooled root material from 4 plates, each containing 12–15 Arabidopsis seedlings. For each time point, different letters indicate statistically significant differences between treatments (Tukey’s test; *P*<0.05).

NO accumulates in plant roots during the early stages of Trichoderma interaction with Arabidopsis and tomato plants (Gupta *et al.*, 2014; Martínez-Medina *et al.*, 2019*a*). To study whether VCs released by Trichoderma also trigger the accumulation of NO in Arabidopsis roots, endogenous NO accumulation was monitored in roots of Arabidopsis Col-0 seedlings in the split-plate assays by using the NO fluorescent probe DAF-2DA. Roots of untreated control plants contained basal levels of NO along the root (Fig. 3A, B). However, roots of VCs-treated seedlings displayed a more intense NO fluorescence at 1 and 2 days after treatment (Fig. 3A, B). The enhancement of NO fluorescence in VCs-treated seedlings was mostly confined to the outermost cell layers (Fig. 3B). Incubation of roots with the NO scavenger cPTIO extinguished the fluorescence induced by Trichoderma VCs, confirming that the DAF-2DA-related fluorescence was due to NO accumulation (Supplementary Fig. S3). In accordance with the enhancement of NO fluorescence by Trichoderma VCs, the NO-responsive gene *PHYTOGB1* (Perazzolli *et al.*, 2004), which encodes Phytoglobin 1, was up-regulated in Arabidopsis roots in response to Trichoderma VCs compared with untreated control plants (Fig. 3C). Together, these observations strongly support that Trichoderma VCs trigger an early burst of NO in Arabidopsis roots.

### NO signalling is required for Trichoderma VCs-induced expression of *MYB72* in Arabidopsis roots

**Fig. 4.**
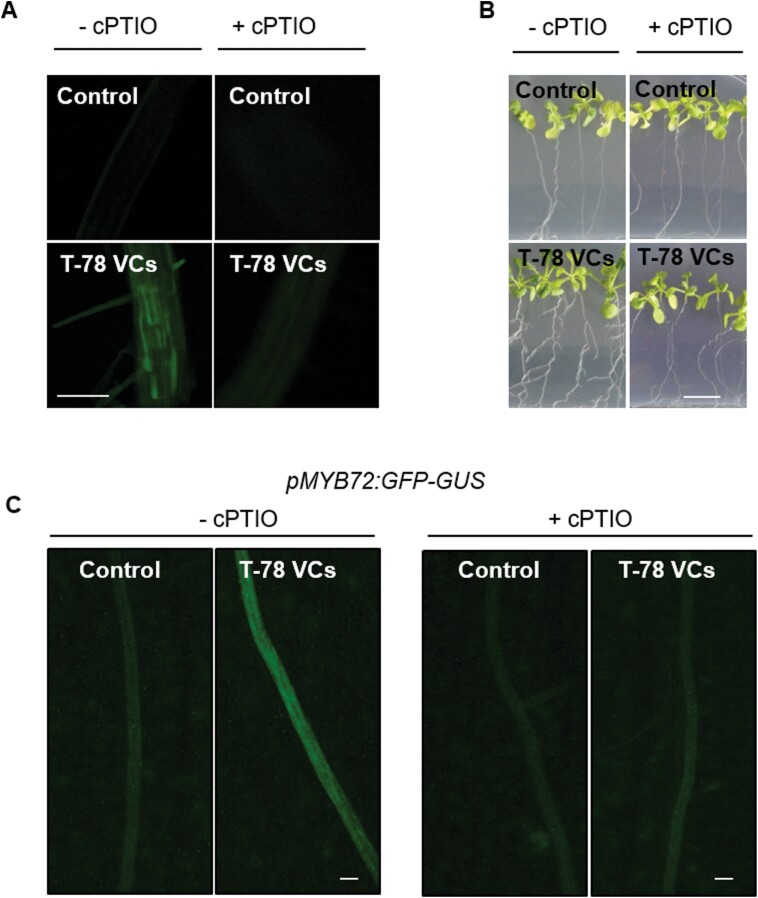
NO accumulation is required for regulation of *MYB72* expression by Trichoderma VCs. (A) NO-dependent DAF-2DA fluorescence (green; excitation at 495 nm, emission at 515 nm) visualized by fluorescence microscopy in roots of Arabidopsis seedlings untreated (control) or treated with VCs from *T. harzianum* T-78 (T-78 VCs) for 2 days in the split-plate assays, supplemented (+cPTIO) or not (-cPTIO) with the NO scavenger cPTIO (500 µM). Scale bar=100 μm. (B) Representative photographs of Arabidopsis seedlings that were untreated (control) or treated with T-78 VCs for 3 days in split-plate assays in the absence or presence of cPTIO (500 µl of 500 µM). Scale bar=1 cm. (C) Representative images showing the accumulation of GFP (green signal) in p*MYB72:GFP-GUS* roots by using fluorescence stereomicroscopy. Seedlings were untreated (control) or treated with T-78 VCs for 2 days in split-plate assays in the absence or presence of cPTIO (500 µM). Scale bar=200 μm. These results are representative of two independent experiments.

We previously found that Trichoderma VCs trigger the expression of *MYB72* in Arabidopsis roots (Martínez-Medina *et al.*, 2017*b*). To determine whether NO signalling is required for Trichoderma VCs-mediated regulation of *MYB7*2, we focused specifically on VCs from T-78, as this isolate has been extensively studied in our laboratory with regard to NO signalling and ISR (Martínez-Medina *et al.*, 2013, 2019*a*). We used the Arabidopsis transgenic line *pMYB72:GFP-GUS*, which expresses the GFP-GUS fusion protein under the control of the *MYB72* promoter (Zamioudis *et al.*, 2015), and a pharmacological approach using the NO scavenger cPTIO. Although the use of cPTIO has some limitations (D’Alessandro *et al.*, 2013), it allows NO depletion regardless of the NO plant source. To assess the suitability of using cPTIO as a NO scavenger in our experimental setup, we first tested the efficiency of cPTIO in reducing NO accumulation triggered by T-78 VCs in Arabidopsis roots in the split-plate assays. cPTIO efficiently prevented NO accumulation (Fig. 4A) and the promotion of lateral roots typically triggered by T-78 VCs (Fig. 4B). As expected, the treatment with T-78 VCs led to a stronger accumulation of the GFP fluorophore in roots of *pMYB72:GFP-GUS* plants compared with control roots (Fig. 4C), confirming that T-78 VCs induce *MYB72* expression in Arabidopsis roots. Interestingly, the induction of *MYB72* gene expression by T-78 VCs was abolished in seedlings treated with cPTIO (Fig. 4C), demonstrating that NO signalling is involved in Trichoderma VCs-mediated regulation of *MYB72* in Arabidopsis roots.

### NO signalling in roots is required for ISR triggered by Trichoderma VCs

**Fig. 5.**
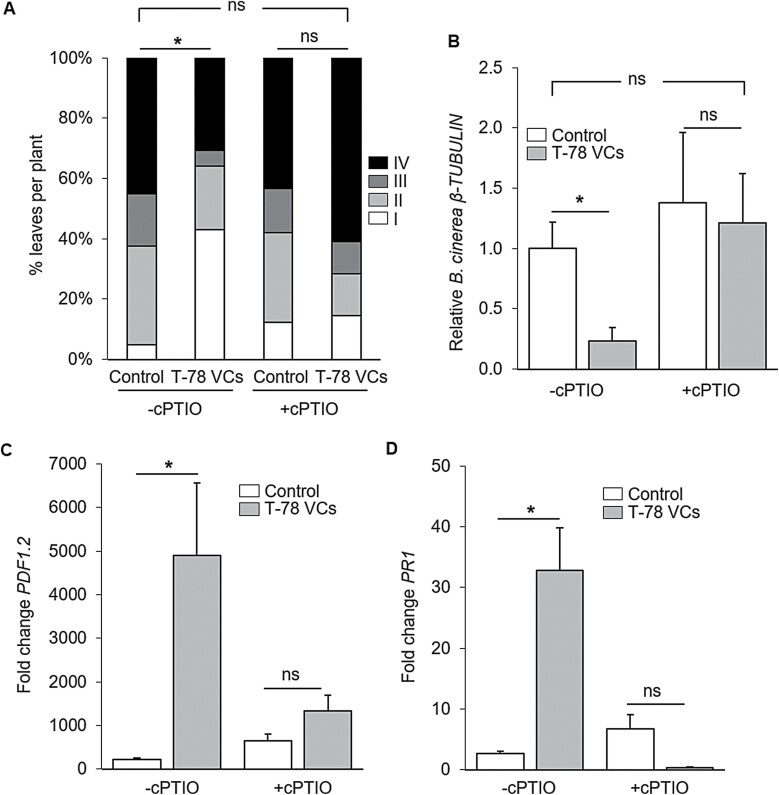
NO signalling is required for Trichoderma VCs-mediated ISR against *B. cinerea*. Quantification of *B. cinerea* disease symptoms (A) and the relative amount of *B. cinerea* (B), and relative expression of the defence-related marker genes *PDF1.2* (C) and *PR1* (D) in leaves of Arabidopsi*s* Col-0, 1 day after inoculation with *B. cinerea*. Seedlings were untreated (control) or treated with VCs from *T. harzianum* T-78 (T-78 VCs) for 3 days in split-plate assays that were supplemented (+cPTIO) or not supplemented (-cPTIO) with cPTIO (500 µl of 500 µM). Subsequently, seedlings were transplanted into pots and 3 weeks later inoculated with *B. cinerea*. In (A), disease severity was scored 3 days after inoculation by using four disease severity classes: I, no visible disease symptoms; II, non-spreading lesion; III, spreading lesion without tissue maceration; IV, spreading lesion with tissue maceration and sporulation of the pathogen. The percentage of leaves in each class was calculated per plant. Asterisks indicate statistically significant differences compared with untreated control plants (χ ^2^ test; α=0.05; *n*=50 plants; ns, not significant). In (B), the relative amount of *B. cinerea* was determined by qRT–PCR analysis of the *B. cinerea β-TUBULIN* gene relative to the Arabidopsis *TUBULIN-4* gene. The expression levels are reported as the fold change relative to that found in control plants not pre-treated with cPTIO. Values are the means ±SE of four biological replicates. In (C) and (D), *PDF1.2* and *PR1* gene expression was normalized to that of the *TUBULIN-4* gene. The expression levels are reported as the fold change relative to that of plants not inoculated with *B. cinerea* in each treatment. Values are the means ±SE of at least four biological replicates. In (B–D), asterisks indicate statistically significant differences (Student’s *t*-test; *P*<0.05). ns, not significant.

Given the critical role of *MYB72* in Trichoderma VCs-mediated ISR (Fig. 1; Martínez-Medina *et al.*, 2017*b*), and the importance of NO signalling in the regulation of *MYB72* by Trichoderma VCs (Fig. 4), we aimed to discern whether NO function in roots is required for VCs-mediated ISR. To this end, Arabidopsis Col-0 seedlings were treated with T-78 VCs for 3 days in split plates and root-supplemented, or not supplemented, with cPTIO. Subsequently, seedlings were transplanted into pots, and 3 weeks later, the plants were challenged with *B. cinerea*. As in the previous experiments, plants exposed to T-78 VCs developed significantly less severe disease symptoms (Fig. 5A), and contained less pathogen biomass, as determined by RT–PCR quantification of *B. cinerea β-TUBULIN* transcript levels (Fig. 5B). Remarkably, plants whose roots were pre-treated with cPTIO did not develop T-78 VCs-mediated ISR against *B. cinerea* (Fig. 5A, B). Similarly, T-78 VCs did not reduce *B. cinerea* symptoms and fungal biomass in the nitrate reductase double mutant *nia1nia2* line, which is impaired in nitrate reductase-dependent NO production (Supplementary Fig S4). Taken together, these observations provide evidence that NO signalling in roots is essential for the ISR triggered by T-78 VCs, and suggest a role for the nitrate reductase pathway as one of the sources of NO involved in the ISR response triggered by T-78 VCs.

### Inhibition of NO accumulation in the roots compromises Trichoderma VCs-mediated defence priming in the leaves

To gain further insight into the role of NO root signalling in Trichoderma VCs-mediated ISR against *B. cinerea*, we assessed the expression of *PDF1.2* and *PR1* in response to *B. cinerea* infection in plants pre-treated, or not treated, with the NO scavenger cPTIO. Arabidopsis Col-0 seedlings were first exposed to T-78 VCs in split plates and root-supplemented, or not supplemented, with the NO scavenger cPTIO. Seedlings were subsequently transplanted into pots and 3 weeks later were challenged with *B. cinerea*. One day after pathogen challenge, we assessed *PDF1.2* and *PR1* expression in the infected leaves. A significantly higher level of expression of *PDF1.2* and *PR1* was observed in *B. cinerea*-challenged plants that had previously been exposed to T-78 VCs compared with untreated control plants (Fig. 5C, D). This enhanced *B. cinerea-*induced expression pattern of *PDF1.2* and *PR1* in T-78 VCs pre-treated plants was abolished in plants whose roots were treated with cPTIO during the T-78 VCs exposure (Fig. 5C, D). These results support a role for root NO in the activation of T-78 VCs-mediated ISR in which priming for enhanced defence-related gene expression is important to combat *B. cinerea* infection.

## Discussion

Selected Trichoderma strains can improve plant health by triggering a broad-spectrum ISR (Segarra *et al.*, 2009; Mathys *et al.*, 2012; Martínez-Medina *et al*., 2013, 2017*a*; Alkooranee *et al.*, 2017; Nawrocka *et al.*, 2018). In Arabidopsis, the root-specific transcription factor MYB72 is essential for the initiation of ISR after root colonization by Trichoderma (Segarra *et al.*, 2009). More recently, it was demonstrated that Trichoderma VCs may act as determinants for the elicitation of *MYB72* expression and ISR via root-to-shoot signalling (Kottb *et al.*, 2015; Martínez-Medina *et al.*, 2017*b*), which is in line with observations obtained with VCs from ISR-eliciting *Pseudomonas* spp. strains (Zamioudis *et al.*, 2015). Here, we first analysed whether MYB72 is also required for the initiation of ISR triggered by VCs released by the ISR-inducing Trichoderma strains *T. harzianum* T-78 and *T. asperellum* T-34. We found that Trichoderma VCs induced resistance against the shoot pathogen *B. cinerea*, and that this phenomenon was associated with primed expression of the JA- and SA-responsive genes *PDF1.2* and *PR1* (Figs 1, 2). It is known that both the SA- and JA-related pathways provide resistance to *B. cinerea* in Arabidopsis plants (Zhang *et al.*, 2017). Indeed, although the JA and SA signalling pathways are usually antagonistic (Hickman *et al.*, 2019, Preprint), the Arabidopsis *coi1-1* and *npr1-1* mutants, which are compromised in JA and SA signalling, respectively, both show a decreased resistance to *B. cinerea*. In analogy, several studies have provided evidence that the JA and SA pathways can function synergistically to mount immune responses (Mur *et al.*, 2006; Zhu *et al.*, 2014; Sharifi and Ryu, 2016; Martinez-Medina *et al*., 2017*a*; Hickman *et al.*, 2019, Preprint). Our results therefore indicate that Trichoderma VCs prime shoot tissues for potentiated SA- and JA-responsive gene expression, conferring enhanced protection against *B. cinerea*. Although we cannot rule out the possible effects of microbially produced CO_2_, which can accumulate in sealed plates (Kai and Piechulla, 2009; Piechulla and Schnitzler 2016), previous studies using non-sealed conditions suggest that other, as yet unknown, Trichoderma VCs must play a role in the observed VCs-stimulated ISR, separately from any role of CO_2_ (Martínez-Medina *et al*., 2017*b*). Interestingly, we found that VCs-mediated ISR and priming against *B. cinerea* was completely abolished in *myb72* plants, supporting that MYB72 is also essential for the activation of ISR by Trichoderma VCs. This finding reinforces the central role of MYB72 in the onset of ISR triggered by different microbes and elicitors.

In Arabidopsis roots, the initiation of the MYB72-dependent iron-deficiency response, which is triggered by *Pseudomonas* spp. as part of the onset of rhizobacteria-mediated ISR, is associated with NO signalling (Zamioudis *et al.*, 2015). Indeed, NO is a well-established key component of the regulatory mechanisms that orchestrate iron uptake in plants (Graziano and Lamattina, 2007; Chen *et al.*, 2010; García *et al.*, 2010; Meiser *et al.*, 2011). We found that plant perception of Trichoderma VCs triggered the expression of *MYB7*2 (Fig. 4), as previously described by Martínez-Medina *et al.* (2017*b*). Interestingly, Trichoderma VCs also triggered a strong accumulation of NO in Arabidopsis roots (Fig. 3), suggesting that NO signalling in roots is an early component of the plant response to Trichoderma VCs. It was previously shown that Trichoderma interaction with Arabidopsis or tomato roots is associated with an early burst of NO (Gupta *et al.*, 2014; Martínez-Medina *et al.*, 2019*a*). Similarly, root perception of rhizobacterial VCs and root interaction with the beneficial fungus *Rhizophagus irregularis* and the pathogenic fungus *Fusarium oxysporum* is also associated with an early burst of NO in roots (Gupta *et al.*, 2014; Zamioudis *et al.*, 2015; Martínez-Medina *et al.*, 2019*b*). These findings suggest that the rapid burst of NO triggered by Trichoderma VCs is part of a common early plant response to microbial elicitors. Remarkably, the NO burst triggered by Trichoderma VCs was mainly restricted to the root epidermis and cortical cells. Similarly, the up-regulation of *MYB72* by Trichoderma and rhizobacteria VCs was found to be restricted to the epidermal and cortical cells (Zamioudis *et al.*, 2015; Martínez-Medina *et al.*, 2017*b*). Thus, NO signalling triggered by Trichoderma VCs is activated in root cell types that are also associated with MYB72-related root responses. By using the NO scavenger cPTIO, we demonstrated that NO signalling in roots is involved in Trichoderma VCs-mediated regulation of *MYB72* expression (Fig. 4). To date, NO has been proposed to modulate the expression of several transcription factors that moderate plant defense responses, for example, selected Arabidopsis SRG (zinc finger transcription factors, *S*-nitrosothiol regulated) and WRKY family members (Parani *et al.*, 2004; Cui *et al.*, 2021). Moreover, NO can also regulate the DNA-binding property of some transcription factors through *S*-nitrosylation (Cui *et al.*, 2021). Although the molecular mechanisms underlying the activity of NO in Trichoderma VCs-mediated regulation of *MYB72* expression remain unknown, our results are in line with those reported by Zamioudis *et al.* (2015), who found that NO is essential for rhizobacterial VCs-mediated induction of *MYB72* during the onset of the iron-deficiency response. Collectively, our findings suggest that NO signalling in roots is involved in the activation of *MYB72* expression triggered by VCs from different microbes, including bacteria and fungi.

**Fig. 6.**
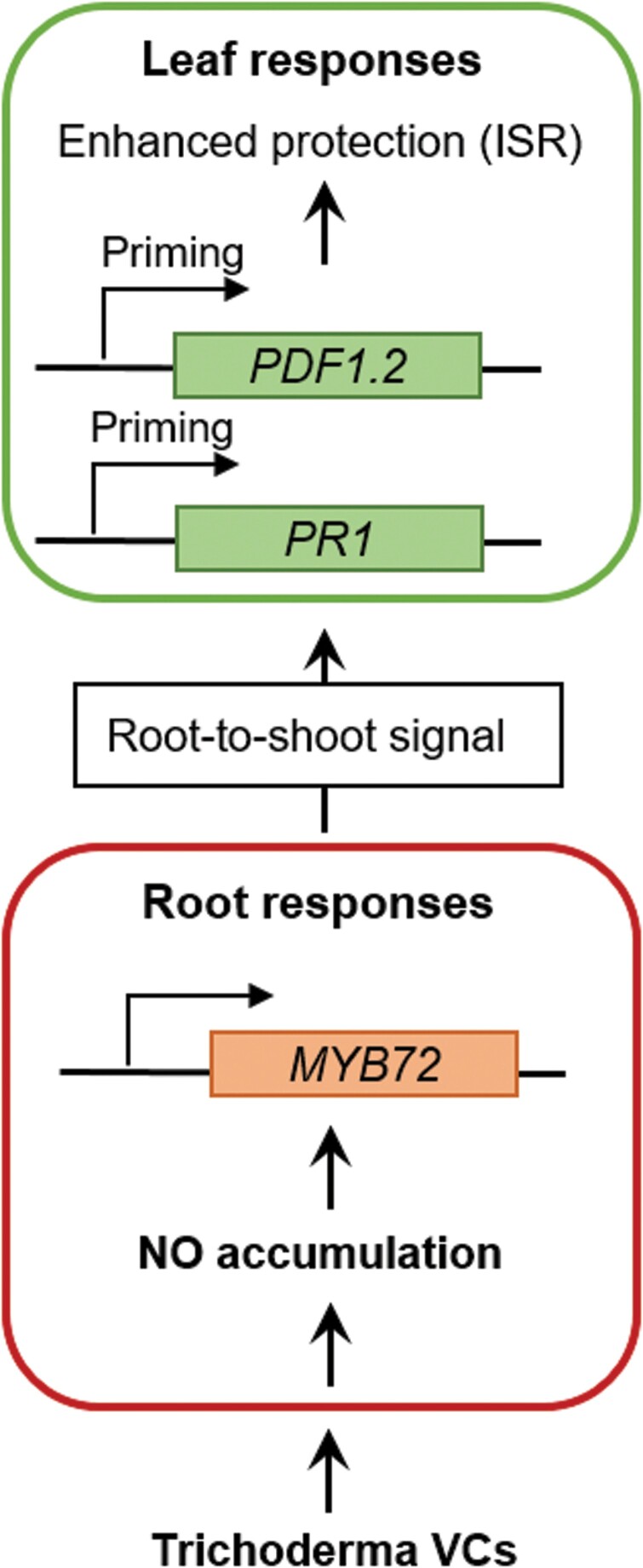
Model for the role of root NO signalling and MYB72 in systemic disease resistance triggered by Trichoderma VCs against *B. cinerea*. Perception of Trichoderma VCs leads to a burst of NO accumulation in the root, which is required for the activation of the root-specific transcription factor MYB72. Subsequently, an as yet unknown root-to-shoot signal is generated, which travels to systemic tissues, priming the leaves for enhanced JA- and SA-regulated defences, and triggering ISR against *B. cinerea* attack.

We found that MYB72 is essential for Trichoderma VCs-mediated priming and ISR in shoots. We further demonstrated that the regulation of *MYB72* expression by Trichoderma VCs was dependent on the accumulation of NO in roots. To analyse the involvement of NO from roots on MYB72-dependent priming and ISR, we performed a bioassay in which NO signalling was compromised in Arabidopsis roots by the use of the NO scavenger cPTIO, specifically during treatment of the plants with Trichoderma VCs. We found that inhibition of the accumulation of NO in roots blocked the VCs-induced priming of *PR1* and *PDF1.2* expression as well as ISR against *B. cinerea* in the leaves (Fig. 5). Thus, our results confirm that NO signalling in roots is essential for the onset of defence priming and ISR triggered by Trichoderma VCs. NO has previously been associated with induced resistance against shoot pathogens, likely by regulating several defence responses, such as the hypersensitive response, callose deposition, *PR* gene expression, and the activity of antioxidant enzymes in the attacked leaves (Manjunatha *et al.*, 2009; Fu *et al.*, 2010; Acharya *et al.*, 2011; Keshavarz-Tohid *et al.*, 2016). Indeed, different transcription factors involved in plant immunity have been described as NO targets (Zimmerli *et al.*, 2000; Conrath *et al.*, 2002; Tada *et al.*, 2008; Lindermayr *et al.*, 2010). Besides this important role of NO signalling in the regulation of leaf defences, we demonstrate here that NO signalling in roots is essential for the onset of the ISR triggered by Trichoderma VCs in leaves. Altogether, our study demonstrates that plant perception of Trichoderma VCs triggers a burst of NO in roots that is required for *MYB72* up-regulation and the priming of defences and activation of ISR in the leaves (Fig 6).

## Supplementary data

The following supplementary data are available at *JXB* online.

Table S1. List of primers used in the analyses.

Fig. S1. Trichoderma VCs trigger the expression of the Trichoderma VCs-responsive genes *FRO2* and *IRT1* in the roots of WT Col-0 and *myb72* Arabidopsis seedlings.

Fig. S2. MYB72 is required for systemic immunity triggered by Trichoderma root inoculation.

Fig. S3. Imaging of NO production in the roots of Arabidopsis treated with Trichoderma VCs and incubated with cPTIO.

Fig. S4. Trichoderma VCs-triggered immunity is abolished in the *nia1nia2* line.

erab294_suppl_Supplementary_Table_S1_Figures_S1-S4

## Data Availability

All data supporting the findings of this study are available within the paper and within its supplementary data published online.
