# Supplementary material for: Nitric oxide signalling in roots is required for MYB72-dependent systemic resistance induced by Trichoderma volatile compounds in Arabidopsis
Source: J Exp Bot. 2021 Jun 15;73(2):584–95. doi: 10.1093/jxb/erab294 (PMC8757496; doi:10.1093/jxb/erab294)
Supplement: erab294_suppl_Supplementary_Table_S1_Figures_S1-S4 [file erab294_suppl_supplementary_table_s1_figures_s1-s4.pdf]

Table S1. List of primers used in the analyses.

ID	Target gene	Sequence (5' → 3')
At5g44420	*PDF1.2*[1]	TTTGCTGCTTTCGACGCAC<br>CGCAAACCCCTGACCATG
At5g24770	*VSP2*[2]	CGGGTCGGTCTTCTCTGTTC<br>CCAAAGGACTTGCCCTA
At2g14610	*PR1*[3]	GGAGCTACGCAGAACAACTAAGA<br>CCCACGAGGATCATAGTTGCAACTGA
XM_001560987.1	*β-TUBULIN B. cinerea*[4]	CCGTCATGTCCGGTGTTACCAC<br>CGACCGTTACGGAAATCGGAA
At5g44340	*TUBULIN-4*[5]	GAGGGAGCCATTGACAACATCTT<br>GCGAACAGTTCACAGCTATGTTCA
At5g09810	*ACTIN7*[6]	AGTGGTCGTACAACCGGTATTGT<br>GATGGCATGAGGAAGAGAGAAAC
At1g01580	*FRO2*[6]	TGTGGCTCTTCTTCTCTGGTGCTT<br>TGCCACAAAGATTCGTCATGTGCG
At4g19690	*IRT1*[6]	ACCCGTGCGTCAACAAAGCTAAAG<br>TCCCGGAGGCGAAACACTTAATGA

[1]Vos *et al*. (2013); [2]Martínez-Medina *et al*. (2017); [3]Journot-Catalino *et al*. (2006); [4]Brouwer *et al*. (2003); [5]Terrón-Camero *et al*. (2020); [6]Zamioudis *et al*. (2015).

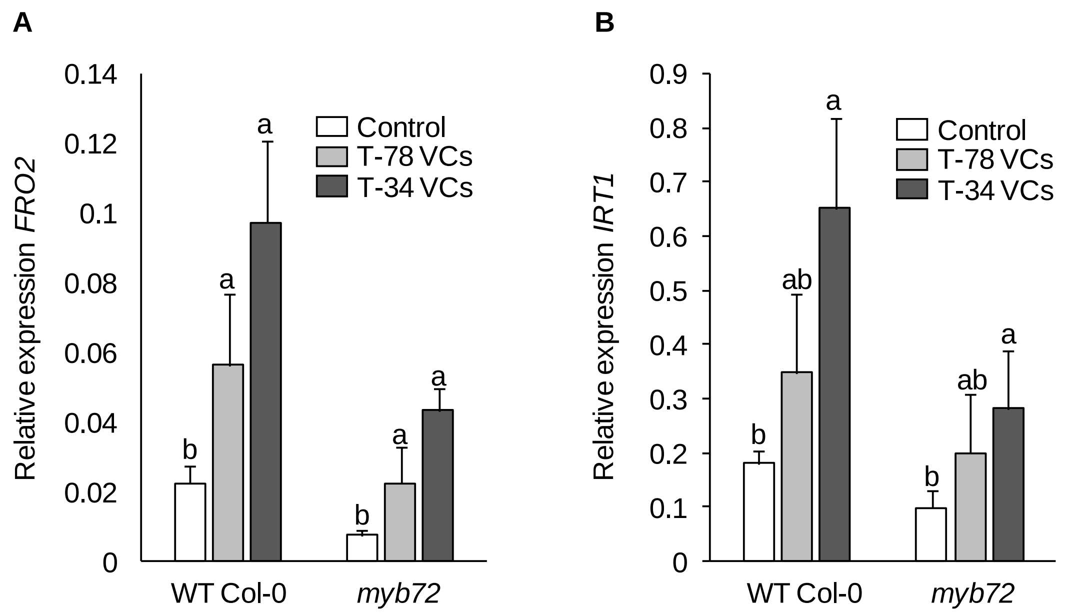

Figure S1. Trichoderma VCs trigger the expression of the Trichoderma VCs-responsive genes *FRO2* and *IRT1* in the roots of WT Col-0 and *myb72* Arabidopsis seedlings. Relative expression of (A) *FRO2* and (B) *IRT1* in roots of Arabidopsis Col-0 and *myb72* mutant seedling that were untreated (control) or treated with VCs from *T. harzianum* T-78 (T-78 VCs) or *T. asperellum* T-34 (T-34 VCs) for 3 days in split-plate assays. Expression was normalized to that of the Arabidopsis *TUBULIN-4* gene. Values are means ± SE of four biological replicates. For each Arabidopsis line, different letters indicate statistically significant differences between treatments (Tukey's HSD test; P < 0.05).

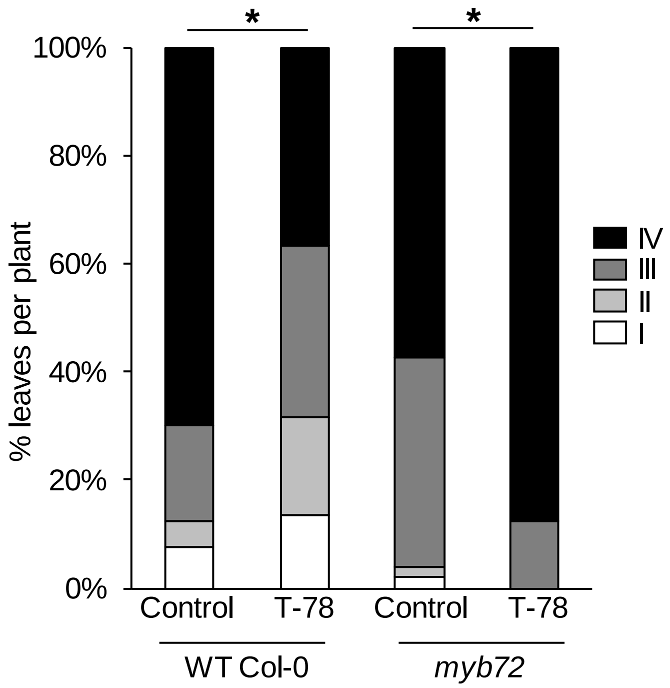

Figure S2. MYB72 is required for systemic immunity triggered by Trichoderma root inoculation. Quantification of disease symptoms in leaves of Arabidopsis WT Col-0 and *myb72* mutant lines after inoculation with *B. cinerea*. Seedlings were untreated (control) or root inoculated with *T. harzianum* T-78 in pots (T-78). The inoculation with T-78 was achieved according to Martínez-Medina *et al.* (2013). Three weeks after transplanting, seedlings were challenged with *B. cinerea*. Disease severity was scored 3 days after inoculation by using four disease severity classes: I, no visible disease symptoms; II, non-spreading lesion; III, spreading lesion without tissue maceration; IV, spreading lesion with tissue maceration and sporulation of the pathogen. Percentage of leaves in each class was calculated per plant. The asterisks indicate statistically significant differences compared with untreated control plants (χ^2 test; $\alpha = 0.05$; $n = 50$ plants).

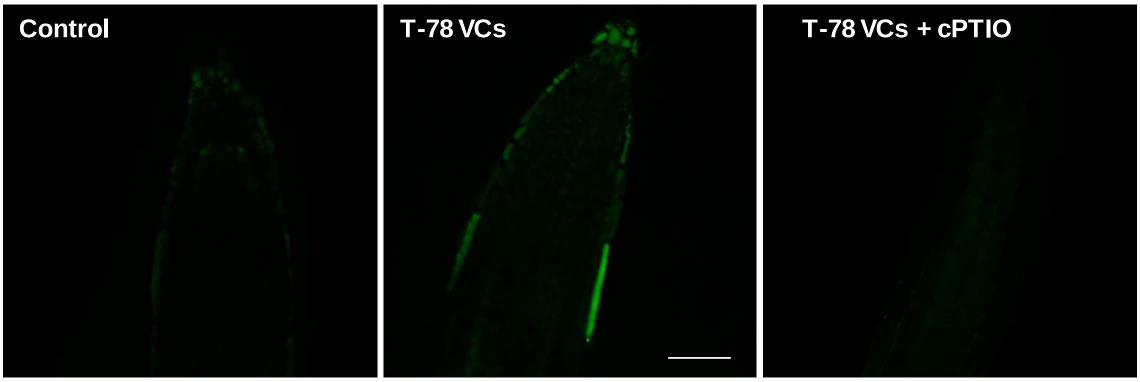

Figure S3: **Imaging nitric oxide (NO) production in the roots of Arabidopsis treated with Trichoderma VCs and incubated with cPTIO.** NO-dependent DAF-2DA fluorescence was visualized by fluorescence microscopy as in Figure 3A, in untreated (control) Arabidopsis Col-0 roots, in roots of Col-0 seedlings treated with VCs from *T. harzianum* T-78 (T-78 VCs) for 2 days using split-plate assays, and in roots of T-78 treated Col-0 seedlings that were incubated with 500 µm cPTIO (T-78 VCs + cPTIO). Scale bar = 50 µm.

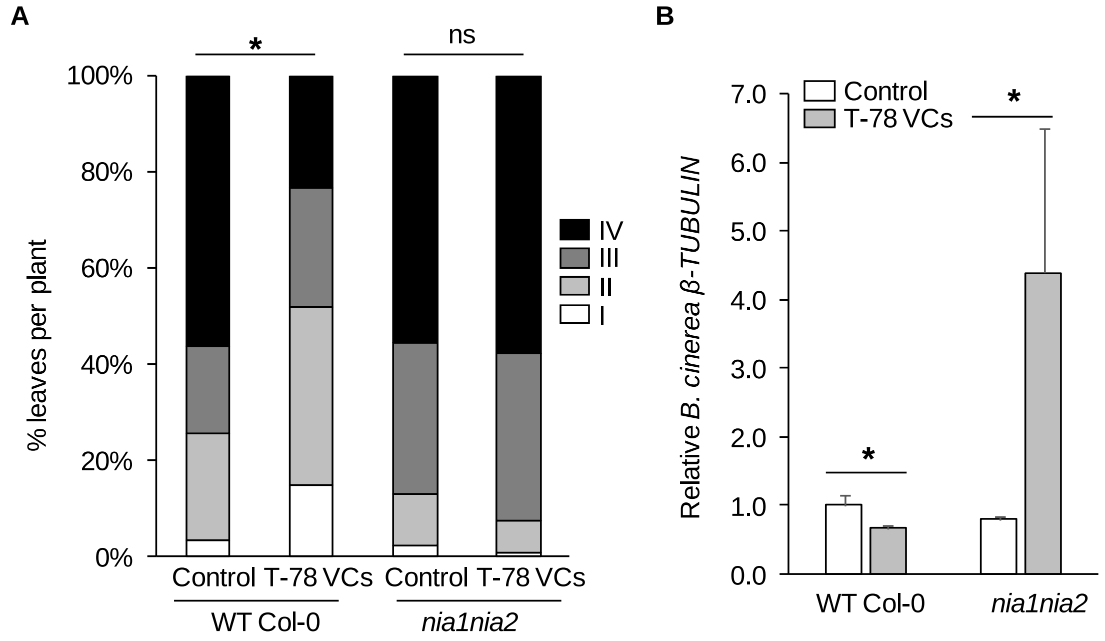

Figure S4. Trichoderma VCs-ISR is abolished in *nia1/nia2* line. (A) Quantification of *B. cinerea* disease symptoms and (B) relative amount of *B. cinerea* in leaves of Arabidopsis WT Col-0 and *nia1/nia2* mutant lines after inoculation with *B. cinerea*. Seedlings were untreated (control) or treated with VCs from *T. harzianum* T-78 (T-78 VCs) for 3 days in split-plate assays before transplanting them into pots. Three weeks after transplanting, seedlings were challenged with *B. cinerea*. In (A) disease severity was scored 3 days after inoculation by using four disease severity classes: I, no visible disease symptoms; II, non-spreading lesion; III, spreading lesion without tissue maceration; IV, spreading lesion with tissue maceration and sporulation of the pathogen. Percentage of leaves in each class was calculated per plant. The asterisks indicate statistically significant differences (χ^2 test; $\alpha = 0.05$; $n = 50$ plants). In (B) the relative amount of *B. cinerea* was determined 2 days after inoculation by quantitative RT-PCR analysis of the *B. cinerea β-TUBULIN* gene relative to the Arabidopsis *TUBULIN-4* gene. The expression levels are reported as the fold increase relative to that found on control Col-0 plants. Values are means ± SE of four biological replicates. The asterisks indicate statistically significant differences according to Student's t-test ($P < 0.05$). ns, not significant.

References

Brouwer M, Lievens B, Hemelrijck W Van, Ackerveken G Van Den, Cammue BPA, Thomma BPHJ. 2003. Quantification of disease progression of several microbial pathogens on *Arabidopsis thaliana* using real-time fuorescence PCR. FEMS Microbiology Letters **228**, 241–248.

Journot-Catalino NJ, Somssich IE, Roby D, Kroj T. 2006. The transcription factors WRKY11 and WRKY17 act as negative regulators of basal resistance in *Arabidopsis thaliana*. The Plant Cell **18**, 3289–3302.

Martínez-Medina A, Fernández I, Sánchez-Guzmán MJ, Jung SC, Pascual JA, Pozo MJ. 2013. Deciphering the hormonal signaling network behind the systemic resistance induced by *Trichoderma harzianum* in tomato. Frontiers in Plant Science **4**, 206.

Martínez-Medina A, Van Wees SCM, Pieterse CMJ. 2017c. Airborne signals by Trichoderma fungi stimulate iron uptake responses in roots resulting in priming of jasmonic acid- dependent defences in shoots of *Arabidopsis thaliana* and *Solanum lycopersicum*. Plant, Cell and Environment **40**, 2691–2705.

Terrón-Camero LC, del Val C, Sandalio LM, Romero-Puertas MC. 2020. Low endogenous NO levels in roots and antioxidant systems are determinants for the resistance of Arabidopsis seedlings grown in Cd*. Environmental Pollution **256**, 113411.

Vos IA, Moritz L, Pieterse CMJ, Van Wees SCM. 2015. Impact of hormonal crosstalk on plant resistance and fitness under multi-attacker conditions. Frontiers in Plant Science **6**, 639.

Zamioudis C, Korteland J, Van Pelt JA, *et al*. 2015. Rhizobacterial volatiles and photosynthesis-related signals coordinate *MYB72* expression in Arabidopsis roots during onset of induced systemic resistance and iron-deficiency responses. The Plant Journal **84**, 309–322.
